# Integrating diagnosis and therapy in hepatocellular carcinoma: advances in nanotheranostics

**DOI:** 10.3389/fbioe.2026.1793834

**Published:** 2026-03-25

**Authors:** Jiansong Shen, Yanyan Wen, Sixue Wu, Wei Tian

**Affiliations:** 1 Division of Hospital Management, Shanxi Medical University, Taiyuan, China; 2 NHC Key Laboratory of Pneumoconiosis, Shanxi Key Laboratory of Respiratory Diseases, Department of Pulmonary and Critical Care Medicine, The First Hospital of Shanxi Medical University, Taiyuan, China; 3 Academy of Medical Sciences, Shanxi Medical University, Taiyuan, China; 4 Department of General Surgery, Shanxi Cardiovascular Hospital, Taiyuan, Shanxi, China

**Keywords:** hepatocellular carcinoma, imaging, nanomaterials, theranostics, tumor targeting

## Abstract

Hepatocellular carcinoma (HCC) poses significant challenges in early diagnosis and effective treatment, leading to a poor prognosis. The emerging strategy of “theranostics,” which integrates diagnostic and therapeutic functions into a single platform, offers a promising solution to overcome these limitations. This review comprehensively summarizes recent advances in the application of nanomaterials for theranostics in HCC. First, we elaborate on the design and construction of nanoplatforms and their unique physicochemical properties, which enable simultaneous imaging (e.g., MRI, CT, fluorescence imaging) and therapy (e.g., chemotherapy, radiotherapy, photothermal therapy, and gene silencing). We then highlight the significant advantages of these nanomaterial-based approaches, such as enhanced tumor-specific targeting, improved imaging sensitivity, reduced systemic toxicity, and synergistic therapeutic outcomes. Furthermore, we critically discuss the current challenges and prospects for the clinical translation of HCC theranostics. This review aims to provide a foundational reference and inspire further innovation in developing efficient nanotheranostic agents for the treatment of HCC.

## Graphical Abstract

**Figure:**
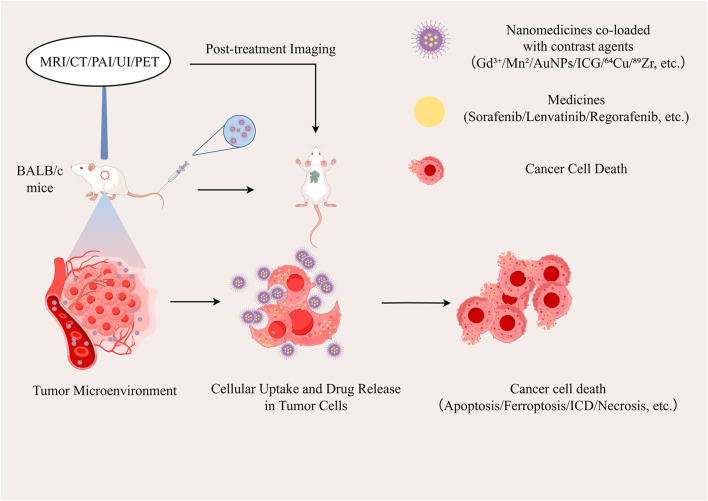
Schematic diagram of the integration of HCC diagnosis and treatment. The graphical abstract was drawn using Figdraw (https://www.figdraw.com), export ID YTSWOff0df. The materials contained in the image are copyrighted by Home for Researchers.

## 1 Introduction

HCC, as the most common primary liver cancer, is ranked sixth among the most commonly diagnosed cancers and the third leading cause of cancer death worldwide (Sung et al., 2021). It has an epidemiological burden in both developing and developed countries (Dimitroulis et al., 2017), with the highest morbidity and mortality in East Asia and Africa (Konyn et al., 2021; Philips et al., 2021). Differences between age and gender are also epidemiological features of HCC, and in most populations, HCC incidence is directly related to age; in most regions, HCC incidence and mortality are higher in men than in women 2–3 times higher (McGlynn et al., 2021). Risk factors for HCC include chronic hepatitis virus infection (HBV/HCV), aflatoxin-contaminated foods, nonalcoholic fatty liver disease, heavy alcohol consumption, obesity, type 2 diabetes, smoking, and metabolic syndrome (Perumpail et al., 2015; Yang et al., 2019; Sagnelli et al., 2020). Major risk factors vary by regions (Akinyemiju et al., 2017; Sung et al., 2021). Although the incidence of HCC has moderated after extensive efforts such as hepatitis B vaccination, hepatitis C treatment regimens, and prevention of aflatoxin exposure, the global incidence and mortality of HCC are still increasing (Singal et al., 2020).

The occurrence of HCC is the result of multiple molecular mechanisms, such as cell cycle disorder, gene instability, and MicroRNA (miRNA) disorder (Aravalli et al., 2008; Jiang et al., 2019; Chidambaranathan-Reghupaty et al., 2021). Therefore, several different cellular phenomena can be observed, such as hypoxia, inflammation, oxidative stress, and tumor microenvironment (TME) (Nishida and Goel, 2011; Aravalli et al., 2013; Alqahtani et al., 2019; Jiang et al., 2019).

Treatment for HCC includes hepatectomy, liver transplantation, ablation, transarterial chemoembolization (TACE), radiotherapy, chemotherapy, and combination therapy. Although hepatectomy/liver transplantation is the single best treatment for HCC and improves survival, up to 70% of patients relapse within 5 years due to tumor microvascular invasion (Llovet et al., 2021). Moreover, most HCC has been clearly diagnosed as advanced and can only be treated with systemic therapy. The conventional chemotherapy drugs of systemic therapy have poor solubility, fast metabolism, low bioavailability, and a lack of specificity. The side effects of chemotherapy drugs are serious, and multi-drug resistance is easy to occur (Kudo et al., 2018; Devan et al., 2021). The above treatment does not achieve satisfactory clinical efficacy, so the development of new therapies is urgently needed to improve this situation.

The abovementioned problems can be solved by nanotechnology-driven drug delivery systems. With the progress of nanotechnology, nanodrug has been widely used in therapy for tumor-targeted and synchronous imaging, and have a remarkable effect in the treatment of HCC. Previous studies have shown that nanomaterials of about 30–200 nm can accumulate more in tumors via enhanced penetration and retention (EPR) effects (Nakamura et al., 2016) or actively targeting. Upon arrival in tumors, nanomaterials effects with specific proteins, mRNA, reactive oxygen species (ROS), acidic conditions in the TME, high glutathione (GSH), and hypoxic stimulation in response (Li et al., 2017). Meanwhile, it can be combined with nanomaterials for magnetic resonance imaging (MRI), positron emission/computed tomography (PET/CT), photoacoustic/ultrasound imaging (PAI/UI) for theranostics and therapeutic effect evaluation.

In 1998, John Funkhouser first proposed the term “theranostics” (Lim et al., 2015). Its connotation is now defined as a combination of molecularly targeted imaging and therapy in which imaging provides actionable information that enables new or more effective therapies (Weber et al., 2023). In recent years, the integration of nanomaterials in the diagnosis and treatment of cancer become a major concern, which has greatly improved the availability of treatment and timeliness of diagnosis. Most importantly, the integration of diagnosis and treatment allows for real-time monitoring of the treatment process and timely feedback of treatment outcomes, thereby providing personalized treatment (Jeyamogan et al., 2021). Here, this review overviews the construction and characteristics of nanomaterials as therapeutic and diagnostic agents, and summarizes the latest progress of integrated diagnosis and treatment of HCC (Table 1). Analyze the advantages of integrated diagnosis and treatment of HCC, as well as the future development potential and challenges of the integrated diagnosis and treatment of HCC.

**TABLE 1:** Application of integrated diagnosis and treatment of hepatocellular carcinoma in this review.

Nanomaterials	Imaging method	Treatment	Application	Ref
HA derivative NPs-DOX-SPIO	MRI	Chemotherapy	Constructed for HCC diagnosis and therapy	Yang et al. (2020b)
SPION-NHs nanocapsule hydrogels	MRI	MHT	For multiple MHT and long-term MRI contrast	Zhang and Song (2016)
FePt@MMT-MIT NPs	MRI	MFH/chemotherapy	For simultaneously visualizing HCC by enhancing MRI signals, treating various diseases, and being used as an inducer of MFH	Chan et al. (2021)
GOD-ESIONs@EVs	MRI	The sequential nanocatalytic Treatment	With efficient targeting capability and catalytic therapeutic efficacy against HCC	Wu et al. (2021)
Polymeric micelle (SPIONs + SF)-AbGPC3	MRI	Chemotherapy	For simultaneous HCC-targeted delivery of SF and tumor detection with MRI	Cai et al. (2020)
Fe@EGaIn/CA - DOX·HCl microspheres	MRI/CT	NIR laser enhanced Chemoembolization	Applications in clinical transcatheter arterial chemoembolization	Wang et al. (2021)
Ce6-SCs	MRI/PAI	PDT	As a magnetic resonance and fluorescence contrast agent	Amirshaghaghi et al. (2019)
IR820-PEG-MNPs	MRI/PAI	PTT	For the precise diagnosis of orthotopic micro HCC and imaging-guided photothermal ablation	Chen et al. (2020)
CSP/TPE@siRNA-SP94 NPs	MRI/FLI	Gene therapy	Developed a theranostic nanoprobe for HCC-targeted gene therapy, and monitored the lesion through dual-imaging modalities	Zhang et al. (2022)
Targeted nanoprobe (SP + Fe3O4 NPs + PEG)	MRI/PAI	PTT	A versatile targeted organic-inorganic hybrid nanoprobe was synthesized as an HCC-specific contrast agent for sensitive and efficient theranostics	He et al. (2021)
(ICG + S) @mSiO2 nanosystem	FLI	PTT/immuno-enhanced therapy	Developed a therapeutic nanosystem for HCC that combines real-time fluorescence imaging, PTT, and immune-enhancement	Yang et al. (2020a)
HCPT@NMOFs-RGD	FLI	Chemotherapy/PDT	Accomplish tumor targeting and play synergistic chemo- and photodynamic therapeutic effects on HCC	Shang et al. (2022)
(DiR + DOX)/DEX-PLA micelles	FLI	Chemotherapy/PTT	For performing image-guided chemo-photothermal therapy	Shi Y. et al. (2021)
CAR-T-MSN-IR780	FLI	PTT	Demonstrated enhanced tumor targeting and anti-tumor capabilities *in vitro* and vivo	Ma et al. (2020)
IRDye800CW-SAHA	FLI	Surgery	Developed an HDAC-targeted fluorescence probe for HCC detection and fluorescence image-guided resection	Tang et al. (2020)
ICG-CD@EPS	FLI/PAI	PTT/Chemotherapy	Provided a feasible and controllable strategy for synergistic PTT and chemotherapy treatments for HCC	Sun et al. (2022)
NGR@DDP	FLI/PAI	SDT/Chemotherapy	Developed a tumor-specific and multiple-stimuli responsive nano-riceball and validated for enhanced sono-chemotherapy	Shi X. et al. (2021)
iRGD-ICG-10-HCPT-PFP-NPs	PAI/UI	Chemotherapy	Nanoparticles with Photoacoustic/Ultrasound Dual-Modality Imaging for against HCC	Li et al. (2021)
Pt@PDA-c NPs	PAI(CT)	PTT	Developed for the early detection of small HCC with NIR-II PACT and for efficient noninvasive photothermal ablation of orthotopic tumors	Qi et al. (2021)
^90^Y/^89^Zr-αGPC3	PET/CT	PTT/PDT/Chemotherapy	Glypican-3 targeted delivery of 89Zr and 90Y as a theranostic radionuclide platform for HCC	Labadie et al. (2021)
Bi/Se - Lenvatinib NPs	CT	SDT	As theranostic nanoparticles for image-guided HCC radiotherapy	Liu J. et al. (2021)
SP94-Fe3O4@ICG&DOX	MPI/FLI	SDT/Chemotherapy	A pH- and photothermal-responsive drug delivery system with good biocompatibility and targeting capability	Jin et al. (2024)
GC-NBs	USI	PTT/SDT/Immunotherapy	An HCC-targeted, stimuli-responsive nanoplatform achieves visualized sonodynamic therapy	Zhang et al. (2024)
C/DCNB	CEUS/USI	PTT/PDT/immunotherapy	Dual-drug loaded nanobubbles combined with sonodynamic and chemotherapy for HCC therapy	Guo et al. (2024)
Ti_3_C_2_/TiO_2_	PA	PTT/SDT/immunotherapy	Novel nanozymes for combined phototherapy and sonodynamic therapy of HCC	Yu et al. (2024)
MPDA/ICG@M1NV	FLI	PTT/immunotherapy	An MPDA/ICG-coloaded exosome-like nanomedicine for synergistic tumor-targeted phototherapy and immunotherapy	Chen et al. (2024)
LA-CMGL	MRI	Chemotherapy/Gene therapy	An MRI-visible, actively targeted chemo-gene co-delivery system for HCC therapy	Rong et al. (2024)

## 2 Classes of nanocarriers

**FIGURE 1:**
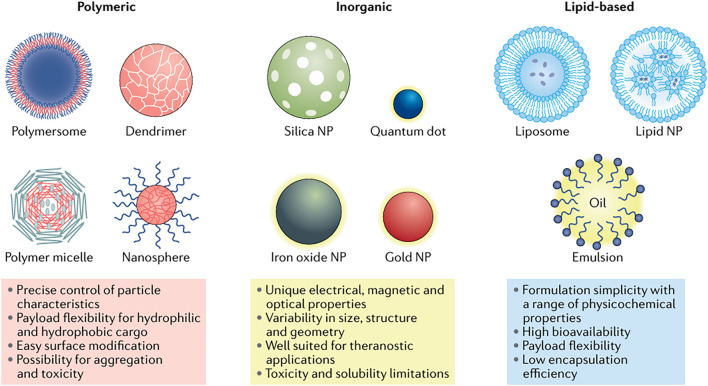
Classes of NPs. Reproduced from Mitchell, M.J. et al. Engineering precision nanoparticles for drug delivery. Nat. Rev. Drug Discov. 20, 101–124 (2021). DOI: 10.1038/s41573-020-0090-8. Reproduced with permission from Springer Nature. Permission conveyed through Copyright Clearance Center, Inc.

The integration of diagnosis and treatment necessitates nanosystems with multifunctional properties that enable simultaneous imaging and therapy. Some nanomaterials can be used for imaging and treatment simultaneously. Other nanomaterials primarily function as nanocarriers carrying imaging agents and therapeutic agents. All these nanocarriers are broadly categorized into organic, inorganic, and organic-inorganic hybrid nanomaterials (Mitchell et al., 2021) (Figure 1).

### 2.1 Organic nanocarriers

Organic nanocarriers are systematically classified into two principal categories based on their structural composition: polymer-derived systems and lipid-based architectures. Polymeric nanocarriers include dendrimers, polymer-based nanoparticles, micelles, gels, drug conjugates, and protein nanoparticles. Lipid nanocarriers include liposomes, exosomes, and solid lipid nanoparticles (Yetisgin et al., 2020). Organic nanocarriers exhibit excellent biocompatibility, negligible cytotoxicity, and improved bio-permeability, rendering them as promising candidates for the development of closed-loop theranostic systems. Organic nanocarriers carrying imaging diagnostic agents and therapeutic agents constitute a complete integrated delivery system for diagnosis and treatment. A polymeric nanomicelle loaded with doxorubicin (DOX) and Purpurin 18 (P18) was encapsulated in erythrocyte membrane-derived vesicles modified with Asn-Gly-Arg (NGR) peptide. NGR peptide can accurately target HCC and provide sonodynamic therapy (SDT) for deep HCC tissue guided by fluorescence imaging/PAI. Organic hyaluronic acid also demonstrates high-affinity targeting of the tumor CD44 receptor. It can carry DOX and superparamagnetic iron oxide (SPIO) to the tumor, and release the drug at the tumor site in response. The redox response of hyaluronic acid in tumors was used for MRI diagnosis and chemotherapy, thus achieving integrated diagnosis and treatment of HCC (Yang et al., 2020b). Hydrogels, characterized by their high water content and porous architecture that closely resembles native tissues, exhibit excellent biocompatibility and typically show low toxicity and gradual degradability under physiological conditions. These properties make hydrogels suitable for a broad range of biomedical applications, including disease therapy, wound healing, and controlled drug delivery (Pablos et al., 2024). Extracellular vesicles (EVs) represent naturally evolved bilayered nanostructures (40–160 nm diameter) with intrinsic membrane-targeting competence, and are ideal organic carriers for the delivery of siRNA or specific proteins (Cheng et al., 2018). Exosomes originating from cancer cells serve as vehicles for circTMEM56, facilitating its delivery to activate the cGAS-STING pathway. This activation ultimately boosts radiation-triggered systemic anti-tumor immune responses, offering a novel approach to enhancing the effectiveness of radiotherapy in treating HCC (Yuan et al., 2025). Extremely small-sized iron oxide nanoparticles (ESION) were first covalently linked to arginine-glycine-aspartate tripeptide (RGD). Then the ESIONs are anchored to EVs by receptor-ligand reactions between RGD and its receptor integrin, and the glucose oxidase (GOD) is loaded onto EVs. When applied to tumors, GOD-functionalized iron oxide nanoparticles (ESIONs) leverage tumor-specific glucose metabolism to generate cytotoxic hydroxyl radicals via Fenton-like reactions, simultaneously enabling T_2_-weighted MRI guidance and mitochondrial-targeted therapy. This dual functionality facilitates real-time treatment monitoring through quantifiable alterations in MRI signal intensity (Wu et al., 2021). Likewise, Organic dihydro porphyrin Chlorin (Ce6) was coated with Superparamagnetic Iron Oxide Nanoparticles (SPION) to form Ce6-SCs, which were clustered in the tumor by the EPR effect. After MRI and PAI detection and verification, Photodynamic therapy (PDT) was performed to realize the integration of diagnosis and treatment (Amirshaghaghi et al., 2019).

### 2.2 Inorganic nanomaterials

Through precise synthetic control, inorganic nanomaterials can be engineered with tailored dimensions, architectures, and morphologies, exhibiting programmable physicochemical properties across electrical, magnetic, and optical domains for multifunctional biomedical applications (Mitchell et al., 2021). Common inorganic nanoplatforms encompass metal-based (gold, iron), semiconductor (quantum dots (QD)), ceramic (silica), and metalloid-derived (arsenic trioxide) systems. These engineered nanostructures readily interface with chemotherapeutic agents through covalent conjugation, π-π stacking, or electrostatic interactions, synergistically enhancing therapeutic outcomes while enabling real-time diagnostic monitoring via their intrinsic optical, magnetic, or radioactive properties. Liu et al. synthesized Bi/Se-Len NPs using selenium (Se) nanoparticles loaded with bismuth (Bi)-QDs and lenvatinib simultaneously. Bi-QDs can be used as a CT contrast agent to guide stereotactic body radiation therapy (SBRT) after imaging, and synergize with Len to achieve radiotherapy sensitization. The results show that both Len and radiotherapy can enhance immune activation, and the combination of Len and radiotherapy synergistically amplifies antitumor immunity. This indicates that Bi/Se-Len NPs have good CT imaging function and synergistic radiosensitizing effect, while inducing antitumor immune response (Liu J. et al., 2021). A functionalized montmorillonite (MMT) nanocomposite with a porous structure was developed, integrated with iron/platinum nanoparticles (FePt NPs) to serve dual roles: enhancing MRI contrast and acting as magnetic hyperthermia agents for visualized HCC therapy (Chan et al., 2021). While silica-based nanomaterials remain widely utilized in HCC theranostics, enabling multimodal imaging (e.g., MRI, photoacoustic, ultrasound) and diverse therapies (chemotherapy, photothermal/photodynamic/sonodynamic treatments), their inorganic composition necessitates careful consideration of potential cytotoxicity and long-term biosafety (Liu Y. et al., 2021).

### 2.3 Organic-inorganic hybrid nanomaterials

**FIGURE 2:**
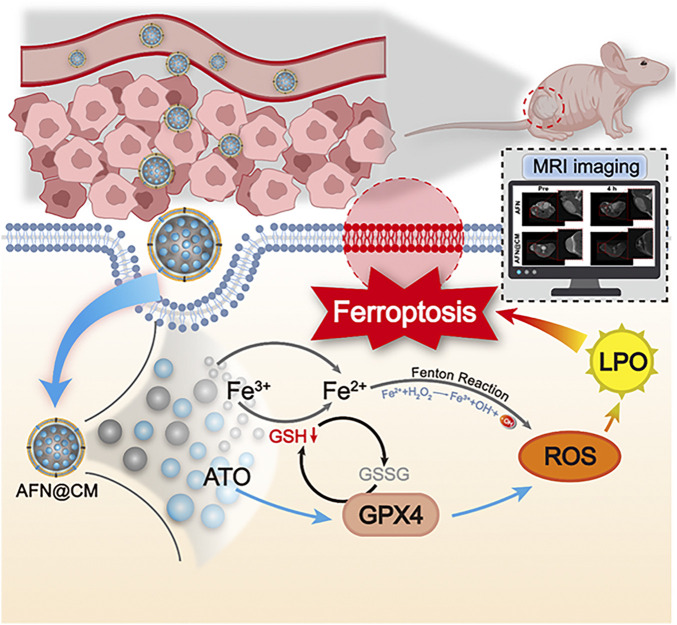
Schematic illustration of the function of AFN@CM. Reprinted from J. Liu et al. Arsenic-Loaded Biomimetic Iron Oxide Nanoparticles for Enhanced Ferroptosis-Inducing Therapy of Hepatocellular Carcinoma. ACS Appl. Mater. Interfaces 2023, *15* (5), 6260–6273. DOI: 10.1021/acsami.2c14962. Copyright 2023 American Chemical Society.

While inorganic nanomaterials exhibit superior structural stability and high drug-loading capacity, they often demonstrate suboptimal biocompatibility profiles and limited biodegradability. In contrast, organic nanomaterials possess inherent biological compatibility but suffer from poor structural integrity and limited functional versatility. To address these complementary limitations, organic-inorganic hybrid nanomaterials have emerged as a transformative strategy, synergistically integrating the advantages of both material classes to create multifunctional therapeutic platforms (Ilhan-Ayisigi and Yesil-Celiktas, 2018; Okubo et al., 2021; Yang et al., 2022). Lee et al. engineered a theranostic nanoplatform by nanoconfining the near-infrared fluorescent dye indocyanine iodide (DIR) within mesoporous silica nanoparticles (MSNs), followed by polyethylene glycol (PEG) surface functionalization to enhance colloidal stability. This innovative design method visualizes the target tissue through real-time fluorescence imaging. Additionally, it exhibits potent photothermal conversion capabilities, efficiently absorbing near-infrared (NIR) radiation and generating heat, which can be harnessed for the photothermal ablation of cancer cells (Lee et al., 2020). Surface modification with organic PEG not only increases stability but also prolongs half-life and reduces immunogenicity and toxicity (Dai et al., 2021). The hybrid organic-inorganic architecture facilitates responsive drug release in the TME by leveraging pH gradients, redox imbalances, or enzymatic activity. This approach allows for precise spatial and temporal control of therapeutic activation, leading to enhanced tumor eradication. For instance, hyaluronic acid copolymers grafted with disulfide-bridged poly-(N-ε-carbobenzyloxy-l-lysine) exhibit redox-sensitive drug release within the TME, enabling the co-delivery of DOX and SPIO nanoparticles for MRI-guided chemotherapy (Yang et al., 2020b). Additionally, arsenic trioxide (ATO) nanoparticles coated with HCC cell membranes have been shown to selectively accumulate in tumors in murine models under MRI monitoring. These biomimetic nanoparticles effectively induce ferroptosis by depleting glutathione and promoting lipid peroxidation, resulting in sustained tumor suppression. This strategy proposes a novel theranostic approach centered on ferroptosis for HCC (Liu et al., 2023) (Figure 2).

Although organic–inorganic hybrid nanomaterials can combine the complementary advantages of both components, several barriers remain. Key concerns include potential toxicity, interfacial compatibility, mechanical stability, tunable and predictable release behavior, scalability, overall biocompatibility, and drug-loading efficiency (Pablos et al., 2024). Before clinical translation, rigorous *in vitro* and *in vivo* studies are essential to establish safety and to define biocompatibility in biologically relevant settings.

## 3 Application of different diagnosis and treatment methods

Current clinical management and investigative approaches for HCC integrate multiple therapeutic modalities, including chemotherapeutic regimens, ionizing radiation protocols, photothermal and photodynamic interventions, sonodynamic treatments, immunotherapies, and genetic modulation strategies. By ingeniously integrating advanced imaging technologies such as MRI, CT, ultrasound imaging, and fluorescence imaging with one or more therapeutic methods onto the same nanomaterials, it is possible to simultaneously achieve precise imaging diagnosis and targeted treatment of HCC, thereby achieving the effect of theranostics.

### 3.1 Chemotherapy/radiotherapy

Chemotherapy remains a fundamental treatment for HCC and is now integrated synergistically into theranostic platforms. Traditional chemotherapeutic medicines such as DOX, paclitaxel (PTX), 10-hydroxycamptothecin (10-HCPT), cisplatin, epirubicin, sorafenib, and lenvatinib primarily exert their anti-tumor effects by disrupting cancer cell proliferation through cytostatic mechanisms (Shewach and Kuchta, 2009). These medicines are increasingly being incorporated into multifunctional nanomaterials, which combine diagnostic imaging and therapeutic capabilities, thus providing the possibility for precise targeted combined treatment strategies for HCC.

**FIGURE 3:**
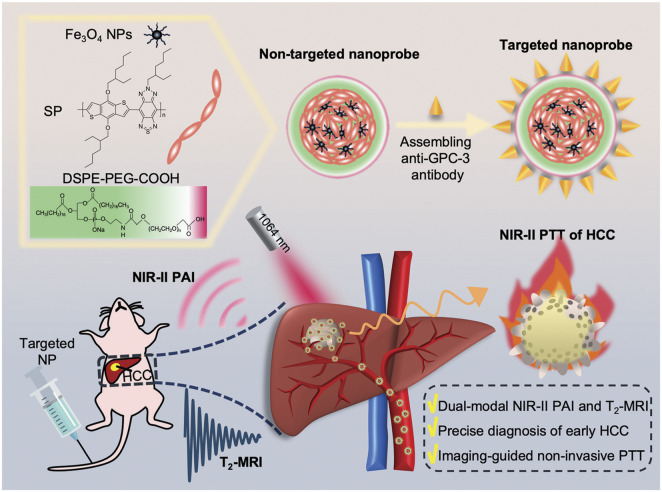
Schematic illustration of the targeted nanoprobe synthesis for dual-modal NIR-II PAI/T2-MRI and imaging-guided non-invasive photothermal therapy in orthotopic early-stage hepatocellular carcinoma. Reproduced from L. He et al., A multifunctional targeted nanoprobe with high NIR-II PAI/MRI performance for precise theranostics of orthotopic early-stage hepatocellular carcinoma. J. Mater. Chem. B, 2021, 9, 8779–8792. DOI: 10.1039/d1tb01729b, with permission from the Royal Society of Chemistry. Permission conveyed through Copyright Clearance Center, Inc.

A reduction and pH dual-sensitive diblock copolymer micelles were prepared, which combined chemotherapy drugs with MRI contrast agents, and realized the integration of diagnosis and treatment of non-invasive detection and chemotherapy based on MRI (Cai et al., 2020). MRI can provide anatomical information and combine with various therapeutic methods and imaging modalities, which have greatly improved the early and accurate diagnosis and treatment of HCC. An organic-inorganic hybrid multifunctional theranostic nanoprobe was developed by combining polyethylene glycol (PEG)-functionalized Fe_3_O_4_ nanoparticles with semiconducting polymers and attached GPC-3 antibodies for targeting early-stage HCC. Both *in vitro* and *in vivo* assessments illustrated that this nanoprobe not only possesses dual-modal near-infrared II (NIR-II) PAI and T_2_-weighted MRI capabilities, but also exhibits significant tumor specificity, high photothermal conversion rate (74.6%), and good biocompatibility. This nanoplatform effectively broke through the depth limitation of the single MRI/PAI mode and achieved image-guided photothermal ablation technology. Therefore, it played an important role in the theranostics of early HCC and could significantly improve the prognosis of high-risk patients (He et al., 2021) (Figure 3).

Chemotherapy drugs can also be combined with Fluorescence imaging and other imaging modalities or treatment methods to form a multimodal diagnosis and treatment platform. Nanomaterials integrating indocyanine green (ICG) and chemotherapeutic payloads demonstrate sustained tumor retention (>48 h) under dual-modal fluorescence/photoacoustic imaging surveillance. This spatiotemporal control enables sequential therapeutic activation: post-accumulation phase near-infrared irradiation (808 nm) triggers photothermal-chemotherapeutic synergy, achieving tumor-specific ablation while minimizing systemic toxicity (Sun et al., 2022).

Meanwhile, ultrasound imaging can be combined with chemotherapy for the integrated diagnosis and treatment of HCC. The method uses an imaging medium with a small particle size to improve the imaging diagnosis effect (Maresca et al., 2018). Li et al. developed tumor-targeting bimodal probes by modifying their surface with iRGD peptides to facilitate deep penetration into tumors. Upon exposure to low-intensity focused ultrasound (LIFU), these probes undergo a liquid-gas phase transition, creating a mechanical blasting effect. This effect, combined with the localized release of 10-HCPT, enhances the precision and effectiveness of therapy against solid tumors (Li et al., 2021). In a similar vein, strategies for addressing multidrug-resistant HCC involve integrating photothermal therapy with MRI guidance. This approach overcomes chemoresistance by enhancing drug penetration through thermal amplification and enables real-time monitoring of treatment progress (Huang et al., 2022).

Radiotherapy synergizes with imaging-guided therapeutic approaches to enhance therapeutic efficacy, mitigate systemic toxicity, and circumvent chemoresistance. Labadie et al. engineered a GPC3-targeted theranostic platform utilizing zirconium-89 (^89^Zr) for PET/CT imaging and yttrium-90 (^90^Y) for radioimmunotherapy (RIT), demonstrating sustained antigen engagement and dosimetric predictability in HCC xenografts. This radioimmunoconjugate system validated the translational potential of real-time treatment response monitoring while advancing diagnostic and therapeutic paradigms (Labadie et al., 2021). Complementarily, bismuth/selenium-lenvatinib nanoparticles (Bi/Se-Len NPs) remodel the immunosuppressive tumor microenvironment through CT-guided radiation sensitization and immunogenic cell death induction, amplifying abscopal effects against radioresistant HCC lesions (Liu J. et al., 2021).

### 3.2 PDT/PTT,PAI

Photodynamic and photothermal therapies (PDT/PTT) represent promising non-invasive modalities for HCCtreatment. These therapies achieve this by moderately penetrating the tissues and precisely and selectively destroying the tumor cells, while minimizing collateral damage to adjacent non-neoplastic tissue. This effectively avoids the risks that may be associated with traditional surgical treatments. At the same time, it can activate the drug release by adjusting the external light source (Cherukuri et al., 2010; Liu et al., 2019a; Sobhani and Samadani, 2021). Integration with imaging modalities enables real-time monitoring of nanomaterial biodistribution and image-guided temporal control of drug release, optimizing therapeutic efficacy through precision-timed intervention.

PDT operates through light activation of photosensitizers to generate cytotoxic ROS, inducing selective cancer cell apoptosis. Current photosensitizer platforms include porphyrin derivatives, aggregation-induced emission (AIE) luminogens, and near-infrared-absorbing compounds (Dolmans et al., 2003). The inherent fluorescence properties of many photosensitizers facilitate simultaneous tumor imaging and therapeutic intervention, enabling real-time treatment monitoring. For instance, a theranostic nanoplatform was engineered through integration of 10-HCPT into porphyrinic metal-organic frameworks (MOFs) functionalized with RGD-targeting peptides (HCPT@MOF-RGD). This system allows for fluorescence image-guided combinatorial chemotherapy and PDT, showcasing improved HCC targeting and synergistic therapeutic effects (Shang et al., 2022). There are also many nanotherapeutic diagnostic probes developed based on AIE photosensitizers and near-infrared photosensitizers for targeted imaging and photodynamic therapy of HCC(Shi et al., 2016; Gao et al., 2019; Siriwibool et al., 2020; Xu et al., 2021; Zhao et al., 2021).

**FIGURE 4:**
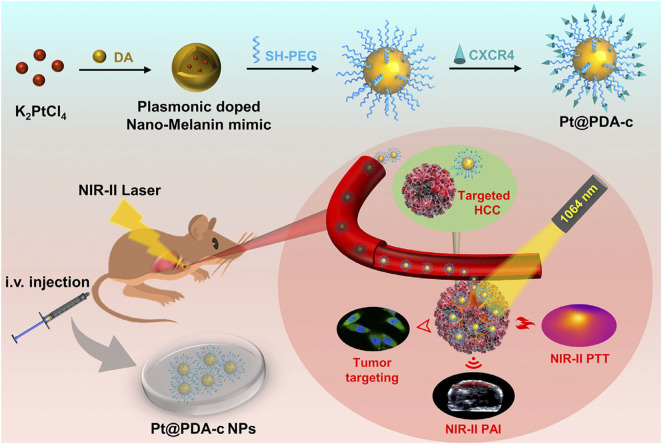
Preparation of Pt@PDA-c nanoparticles and their applications in PACT-guided PTT for orthotopic HCC-bearing nude mice. Reprinted from Qi S et al., “Plasmonic-doped melanin-mimic for CXCR4-targeted NIR-II photoacoustic computed tomography-guided photothermal ablation of orthotopic hepatocellular carcinoma,” Acta Biomaterialia, vol. 129, pp. 245–257 Jul. 2021, DOI: 10.1016/j.actbio.2021.05.034, with permission from Elsevier.

PTT is primarily directed by PAI, with multimodal integration facilitating accurate HCC theranostics by enabling real-time visualization of tumor margins and therapeutic response (Wu et al., 2020). The current development of photothermal agents (PTA) has expanded to include near-infrared dyes such as ICG, IR780, and IR820 derivatives, which also function as contrast agents for PAI due to their dual capabilities in generating photothermal and acoustic signals (Zheng et al., 2018; Jin et al., 2024). PAI combines the high sensitivity inherent to optical imaging with enhanced tissue penetration capabilities, facilitating non-invasive structural visualization of tumor vasculature and dynamic tracking of nanocarrier biodistribution during therapeutic interventions (Wang and Hu, 2012; Liu et al., 2019b). An innovative strategy integrates high-resolution NIR-II photoacoustic computed tomography (PACT) with image-guided PTT for *in situ* small HCC management. For instance, Platinum-doped plasma polydopamine nanoparticles not only enhance the spatial resolution through PACT to enable quantitative and real-time tracking of sub-centimeter-sized HCC, but also possess efficient photothermal conversion capabilities, which can precisely ablate the liver cancer lesions while effectively protecting the adjacent healthy liver tissues (Qi et al., 2021) (Figure 4). The near-infrared dye IR820 has been widely applied. Its significant near-infrared absorption can generate a high photoacoustic signal-to-noise ratio and efficient photothermal conversion, thereby facilitating local tumor ablation. In preclinical studies, IR820-PEG-MNPs exhibited exceptional PA contrast capabilities, allowing detection of sub-2 mm *in situ* hepatic tumors during image-guided therapy, while maintaining the integrity of surrounding parenchymal tissue (Chen et al., 2020). Nanomaterials containing indocyanine green (ICG + S) @ mSiO_2_ and micelles loaded with DiR (DiR + DOX)/DEX-PLA were used in the comprehensive diagnosis and treatment research of HCC under fluorescence imaging guidance (Yang et al., 2020a; Shi Y. et al., 2021). In parallel, CAR-T cell membrane-pretended biomimetic nanoparticles were designed to achieve photothermal ablation of HCC under fluorescence navigation (Ma et al., 2020).

**FIGURE 5:**
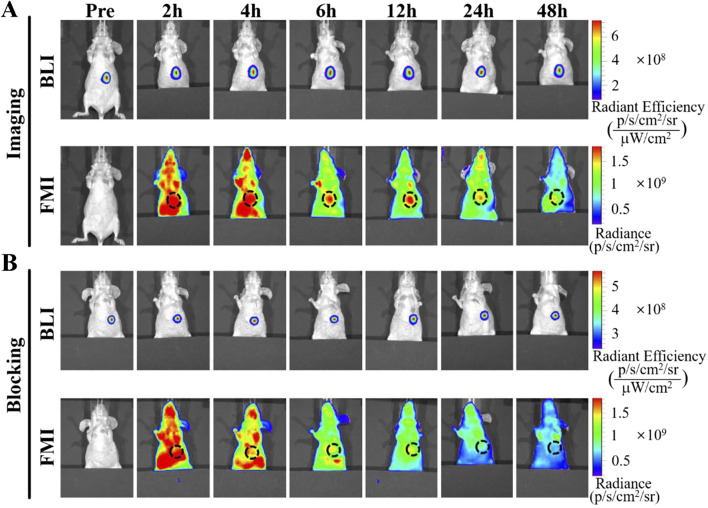
Representative fluorescence molecular imaging (FMI) of IRDye800CW-SAHA on the Bel-7402 orthotopic tumor-bearing mice. **(A)** The targeted imaging of IRDye800CW-SAHA. **(B)** The blocking study with excess HDAC inhibitor SAHA. Reproduced from “Development of a Novel Histone Deacetylase-Targeted Near-Infrared Probe for Hepatocellular Carcinoma Imaging and Fluorescence Image-Guided Surgery,” by C. Tang et al., Molecular Imaging and Biology, 2020, *22*(3), 476–485. DOI: 10.1007/s11307-019-01389-4. Reproduced with permission from SNCSC.

Furthermore, multimodal theranostic agents have been used as embolic agents. Fe@EGaIn/CA microspheres loaded with DOX HCl are used as therapeutic agents for CT and MRI dual-modal imaging-guided and NIR laser-enhanced chemoembolization, and in the near future. Infrared laser-enhanced chemoembolization in auricular tumor-bearing rabbits provides a new direction for multifunctional therapeutic agents (Wang et al., 2021). The integration of NIR fluorescence imaging into surgical interventions now enables real-time intraoperative visualization of HCC, significantly improving surgical precision through improved tumor margin delineation (Nakaseko et al., 2018). The probe IRDye800CW- hexanoyl phenylamine hydroxamic acid (SAHA) targeting (histone deacetylase) HDAC can achieve the optimal ratio of tumor signal to background signal within 6–12 h after injection. Preclinical studies have also confirmed its effectiveness in fluorescence-guided resection of HCC, enabling complete tumor removal while preserving most of the surrounding parenchymal tissue. This advancement is an important step towards image-guided surgical theranostics (Tang et al., 2020) (Figure 5).

### 3.3 Sonodynamic therapy

**FIGURE 6:**
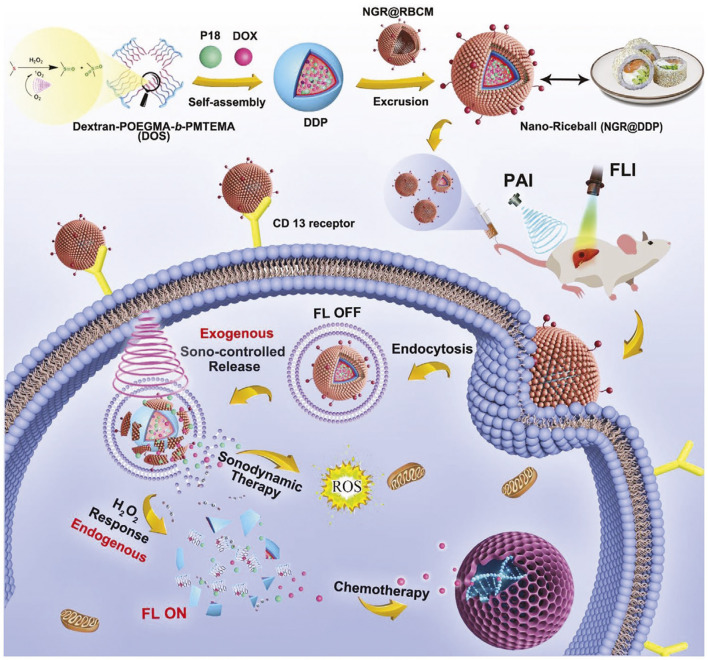
The schematic diagram of tumor-specific and multiple-stimuli responsive nano-riceball (NGR@DDP) for synergistic sono-chemotherapy. Reproduced from “Multi-Responsive Bottlebrush-Like Unimolecules Self-Assembled Nano-Riceball for Synergistic Sono-Chemotherapy,” by G. Liu et al., Small Methods 2021, *5*, 2000416. DOI: 10.1002/smtd.202000416. Reprinted with permission from John Wiley and Sons. Permission conveyed through Copyright Clearance Center, Inc.

Sonodynamic therapy (SDT) is the use of low-intensity ultrasound to penetrate deeply into tissue, based on sonosensitizers in conjunction with sono-chemotherapy (Chen et al., 2014; Zhang et al., 2024). Integrated with fluorescence/photoacoustic imaging, SDT demonstrates exceptional potential for precision theranostics in deep-seated HCC. Nanomaterials (NGR@DDP) loaded with DOX and a sonosensitizer (Purpurin 18, P18) can precisely target HCC cells and undergo ultrasonic dynamic chemotherapy under the guidance of fluorescence imaging/photothermal imaging to treat deep HCC tissues (Shi X. et al., 2021) (Figure 6). Furthermore, the researchers also developed curcumin/doxorubicin nanobubbles (C/DCNB) that can accurately locate HCC. The contrast-enhanced ultrasound (CEUS)-triggered nanobubbles would enhance the permeability of tumor blood vessels, thereby enhancing the EPR effect of the tumor and causing the nanobubbles to accumulate at the tumor site. After being internalized, the drug load is released, and curcumin generates ROS under ultrasound conditions. Moreover, curcumin enhances the sensitivity of tumor cells to doxorubicin by inhibiting the expression of P-glycoprotein. Both *in vitro* and *in vivo* studies have shown that C/DCNB not only promotes ultrasound contrast imaging but also simultaneously delivers therapeutic drugs for imaging and treatment purposes, achieving significant effects (Guo et al., 2024).

### 3.4 Immunotherapy

Immunotherapy is a method of anti-tumor treatment by activating the body’s immune system, using immune checkpoint inhibitors, monoclonal antibodies, vaccines, and adoptive cell therapy, as well as a combination of physical therapies to modulate the immune microenvironment to achieve anti-tumor goals (Sangro et al., 2021; Yu et al., 2024). For example, an intelligent exosome-like nanopharmaceutical was engineered to improve NIR synergistic photoimmunotherapy for HCC. These exosome-like nanoparticles effectively boost the anti-tumor effectiveness by inducing a shift from M2 to M1 phenotype in tumor-associated macrophages (TAMs), consequently reducing the immunosuppressive TME. This modulation enhances the anti-tumor immune responses triggered by immunogenic cell death (ICD) in tumor cells (Chen et al., 2024). The multifunctional (ICG + S)@mSiO_2_ nanosystem integrates fluorescence imaging-guided photothermal therapy with immunomodulation, enhancing CD8^+^ T cell-derived interferon-γ (IFN-γ) secretion and expanding effector memory T cell (TEM), central memory T cells (TCM), and natural killer (NK) cell populations in tumor and splenic microenvironments. This augmentation in cell frequency indicates a potent immune response and relapse prevention activity (Yang et al., 2020a). Additionally, PET/CT-guided radioimmunotherapy platforms demonstrate translational potential for HCC theranostics by synergizing radiolabeled antibody targeting with real-time metabolic imaging (Labadie et al., 2021). Furthermore, adoptive cell therapy incorporates CAR-T cells (Ma et al., 2020) and the immune checkpoint inhibitor PD-L1 for enhanced therapeutic outcomes (Xu et al., 2021).

### 3.5 Gene therapy

**FIGURE 7:**
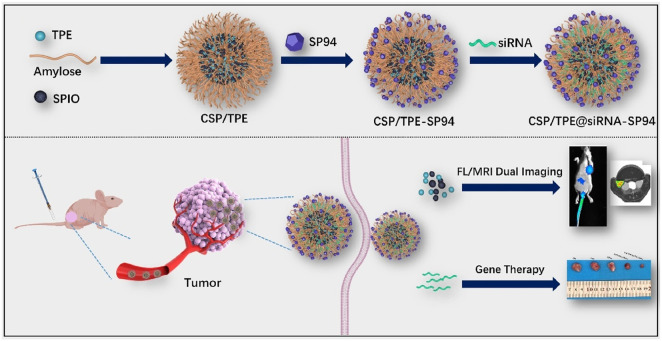
Schematic illustration of the preparation and application of CSP/TPE@siRNA-SP94. Reprinted from H. Zhang et al., “Enhanced fluorescence/magnetic resonance dual imaging and gene therapy of liver cancer using cationized amylose nanoprobe,” Mater. Today Bio, 2022, *13*, 100220, DOI: 10.1016/j.mtbio.2022.100220, with permission from Elsevier.

Gene therapy, an emerging therapeutic strategy, employs gene-editing or delivery systems to correct genetic abnormalities or aberrant protein expression in anti-tumor treatment. In this context, CRISPR/Cas nanotechnology has been developed as a precise genome-editing tool for HCC therapy (Kong et al., 2021). Furthermore, its integration with imaging modalities enables theranostic platforms that unify diagnostic and therapeutic functions. Recently, the integration of chemotherapy-gene combination therapy and MRI-guided diagnosis for treating liver cancer has been achieved through the use of lipid nano-delivery systems or inorganic nano-frameworks (Cullis and Hope, 2017; Rong et al., 2024). These systems carry chemotherapeutic drugs and deliver siRNA, miRNA, and other genes, offering promising strategies for liver cancer treatment. A theranostic nanoprobe designed for targeted gene therapy in HCC involved the conjugation of SP94 peptides to self-assembled nanospheres comprising cationized amylose (CA), SPIO nanoparticles, and tetraphenylene (TPE). These resulting nanospheres (CSP/TPE) were loaded with small interfering RNA (siRNA), and their biodistribution was assessed via dual-modality fluorescence imaging (*in vivo*/*in vitro*) and MRI. The therapeutic efficacy of siRNA was observed in tumors, demonstrating the multifunctional platform that integrates MRI-fluorescence imaging with gene therapy for the diagnosis and treatment of HCC(Zhang et al., 2022) (Figure 7). Furthermore, gene therapy also serves as a supplementary tool in combating liver cancer. For instance, SLC7A11-i, a small interfering RNA (siRNA) directed against the SLC7A11 gene, functions by suppressing SLC7A11 protein expression via gene silencing. This action disrupts cystine uptake and GSH synthesis pathways in HCC, ultimately promoting ferroptosis (Wang et al., 2024).

Nanomedicines supported by versatile carriers and flexible functional design, offering new ways to overcome the limits of conventional HCC treatment. However, much of the field relies on animal models that do not fully capture the complexity of the human tumor microenvironment, so strong preclinical results do not always translate to patients. At the same time, scaling up these formulations is rarely straightforward. Manufacturing can be intricate, and quality standards are not consistent across groups, which slows clinical uptake. The lack of a widely accepted and rigorous framework for evaluating efficacy and safety also makes it difficult to compare studies or integrate findings into a coherent evidence base. Even so, nanomedicine remains a promising path for reshaping HCC care, particularly by advancing theranostic strategies that link diagnosis and treatment while aiming for safer and more effective options for patients.

## 4 Advantages of integrated diagnosis and treatment

### 4.1 Real-time monitoring of the treatment process

**FIGURE 8:**
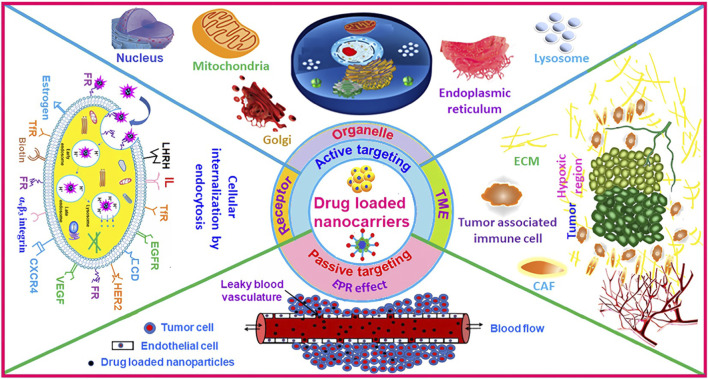
Schematic diagram of nanomaterials for anticancer drug delivery by cancer cell surface targeting, organelle specific targeting, and TME targeting strategies. Reprinted from Dutta, B et al., “Recent advances in active targeting of nanomaterials for anticancer drug delivery,” Advances in Colloid and Interface Science, 2021, *296*, 102509, DOI: 10.1016/j.cis.2021.102509, with permission from Elsevier.

In the treatment of HCC, therapeutic efficacy is directly influenced by drug distribution at the tumor site. Variations in permeability across different regions of the tumor result in uneven distribution of nanomaterials, leading to localized aggregation and limited penetration into the central, highly carcinogenic areas, thereby increasing the risk of recurrence, underscoring the critical need for nanomedicine accumulation in these regions. This targeting capability primarily depends on tumor-specific physiological features, including aberrant vasculature, elevated interstitial pressure, hypoxia, acidic microenvironments, and heightened GSH levels (Minchinton and Tannock, 2006; Tang et al., 2021). Hydroxyapatite (HA) nanomaterials loaded with DOX and SPIO achieved redox-responsive drug release at the tumor site, and demonstrated dual efficacy in anti-tumor therapy and MRI diagnostics (Yang et al., 2020b). These nanomaterials primarily accumulate at tumor sites through the EPR effect, driven by vascular abnormalities, or via active targeting strategies. Active targeting is achieved by functionalizing nanocarriers with ligands like antibodies, proteins, or peptides, to improve tumor-specific accumulation (Dutta et al., 2021) (Figure 8).

However, heterogeneity in vascular permeability across tumor regions leads to uneven distribution of nanomaterials, resulting in localized aggregation and limited penetration into the highly malignant core areas, thereby increasing the risk of recurrence. To overcome this limitation, adjuvant strategies such as hyperthermia employ magnetic-, light-, or radiation-responsive materials to generate localized heating, which disrupts tumor integrity and enhances the penetration of nanomedicines while reducing systemic toxicity (Vilas-Boas et al., 2020; Carter et al., 2021). An injectable, biodegradable nanocapsular hydrogel co-loaded with thermosensitive components and superparamagnetic iron oxide nanoparticles (SPION-NHs) was developed. After four rounds of magnetic hyperthermia (MHT), the xenograft mouse model showed notable tumor suppression without observable harm to adjacent healthy tissues. Additionally, longitudinal monitoring of therapeutic efficacy via T2-weighted MRI over a 21day period validated enduring therapeutic results (Zhang and Song, 2016).

**FIGURE 9:**
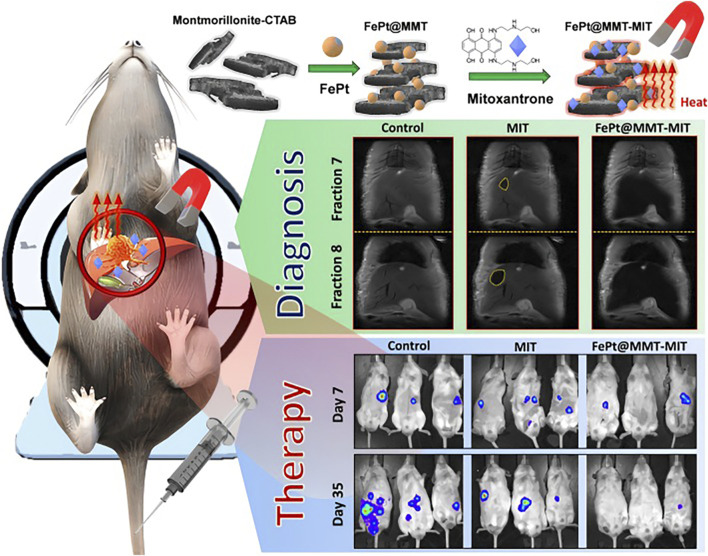
Schematic illustration of how to prepare FePt@MMT-MIT and its diagnosis and treatment of HCC. Reproduced from “Magnetically guided theranostics: montmorillonite-based iron/platinum nanoparticles for enhancing *in situ* MRI contrast and hepatocellular carcinoma treatment,” by Chan, M.H. et al., J. Nanobiotechnol. 2021, *19*, 308. DOI: 10.1186/s12951-021-01052-7. Reprinted with permission from Springer Nature. Permission conveyed through Copyright Clearance Center, Inc.

**FIGURE 10:**
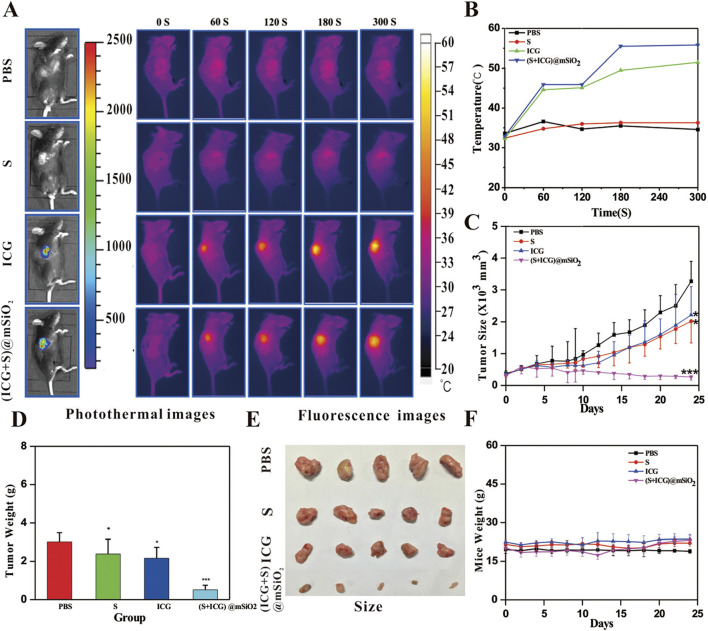
Imaging and synergetic therapy of (ICG+S)@mSiO2 *in vivo*. **(A)** Fluorescence and photothermal imaging of PBS, S, ICG@mSiO2, and (ICG+S)@mSiO2 groups in vivo. **(B)** Temperature rising trend of four groups under 808 nm laser. **(C)** Changes in tumor size in four groups within 25 days (n = 5). **(D)** Changes in tumor weight in four groups after 25 days (n = 5). **(E)** Representative photographs of tumors in four groups. **(F)** Changes in body weight in four groups during 25 days (n = 5). Reproduced from “An NIR-responsive mesoporous silica nanosystem for synergetic photothermal-immunoenhancement therapy of hepatocellular carcinoma,” by Yang, H. et al., J. Mater. Chem. B, 2020, 8, 251-259. DOI: 10.1039/c9tb01891c, with permission from the Royal Society of Chemistry. Permission conveyed through Copyright Clearance Center, Inc.

Clinical imaging modalities such as MRI, PET/CT, and PAI/UI enable precise pathological visualization, facilitating quantitative analysis of nanotherapeutic accumulation at tumor sites. Subsequently, montmorillonite-based iron/platinum nanoparticles (FePt@MMT) were engineered and integrated with chemotherapeutic agents and MRI-guided targeting to construct a multifunctional drug delivery system. This platform enabled real-time MRI monitoring of nanomaterial distribution at tumor sites and simultaneous visualization of therapeutic progression (Chan et al., 2021) (Figure 9). To minimize systemic toxicity and enhance therapeutic precision of anticancer agents, researchers have developed stimulus-responsive modalities, including PDT, PTT/PAT, SDT (Liu et al., 2019a; Son et al., 2020; Sobhani and Samadani, 2021). These approaches leverage external stimuli to induce ROS generation or localized thermal effects, enabling controlled tumor cell elimination. Hence, real-time monitoring of nanotherapeutic distribution at tumor sites is crucial for determining the optimal timing for triggered drug release to enhance therapeutic efficacy. The (ICG + S)@mSiO_2_ were internalized by H22 cells for fluorescence imaging, and fluorescence imaging-guided 808 nm NIR irradiation achieved effective photothermal tumor ablation. Furthermore, the system exhibited immune-potentiating effects (Yang et al., 2020a) (Figures 10A,B). The incorporation of nanotherapeutic targeting and imaging capabilities allows for real-time monitoring of nanotherapeutic biodistribution and treatment dynamics in cancer therapy.

### 4.2 Timely feedback on treatment effect

**FIGURE 11:**
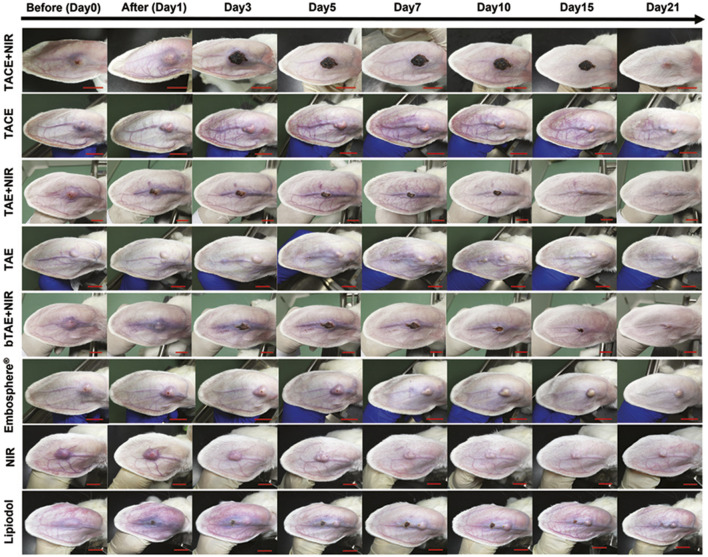
Photographs of tumor growth under different treatment modes. Scale bars: 2 cm. Reproduced from “Magnetic liquid metal loaded nano-in-micro spheres as fully flexible theranostic agents for SMART embolization,” by Wang, D. et al., Nanoscale, 2021, 13, 8817–8836. DOI: 10.1039/d1nr01268a, with permission from the Royal Society of Chemistry. Permission conveyed through Copyright Clearance Center, Inc.

Post-nanotherapeutic intervention, real-time assessment of HCC treatment response facilitates dynamic adjustment of therapeutic regimens based on disease progression. In preclinical studies, therapeutic efficacy is quantitatively assessed through volumetric tumor analysis. A multimodal theranostic platform was engineered by incorporating magnetic liquid metal nanoparticles (eutectic gallium-indium nanodroplets embedded with iron nanoparticles, Fe@EGaIn NPs) encapsulated within calcium alginate microspheres (Fe@EGaIn/CA). This system was modified with doxorubicin hydrochloride (DOX·HCl) to serve as an embolic agent for dual-modality CT/MRI-guided chemoembolization. NIR laser irradiation enhanced therapeutic efficacy through photothermal activation, achieving complete tumor suppression (100% growth inhibition) in auricular tumor-bearing rabbit models, demonstrating optimal treatment outcomes (Wang et al., 2021) (Figure 11). Therapeutic application of (ICG + S)@mSiO_2_ in HCC resulted in a notable reduction in tumor volume and the body weight of four groups scarcely showed any fluctuation (Yang et al., 2020a) (Figures 10C–F). However, clinical translation requires alternative efficacy assessment methods beyond volumetric analysis. Emerging strategies exploit distinct TME characteristics such as acidosis and hypoxia, which markedly differ from normal tissue physiology. Modulating these parameters post-treatment is associated with therapeutic effectiveness. Integrating pH- and oxygen-responsive molecular probes with nanomedicines allows real-time monitoring of treatment efficacy, providing valuable feedback mechanisms for clinical applicability (Fu et al., 2018).

Although many nanocarrier-based delivery systems demonstrate marked tumor-suppressive effects in murine models of HCC, these benefits often fail to translate reliably to humans. One major reason is the substantially greater heterogeneity of human HCC: differences in vascular supply patterns and immune microenvironments are pronounced, leading to wider interpatient variability in therapeutic response even when the same regimen is applied. In addition, many patients present with cirrhosis or fibrosis, which remodels vascular architecture and stromal composition. Such changes can hinder nanoparticle entry into and retention within tumor regions, while also reshaping the kinetics of cellular uptake and drug release. The uncertainty of the EPR effect in clinical settings is also frequently underestimated. Murine tumors typically grow rapidly, exhibit highly permeable vasculature, and show relatively limited variability across individuals. In contrast, human tumors differ substantially in vessel maturity, stromal remodeling, and lymphatic drainage, making “passive accumulation” closer to a probabilistic outcome than a consistent rule.

## 5 Patents and clinical trials

A comprehensive list of patents on drug-loaded nanoparticles for the treatment of HCC was searched using Google Patents search databases and compiled, as shown in Table 2.

**TABLE 2:** A catalog of patents pertaining to nanoparticle-based drug delivery systems for HCC therapy.

Title	Patent No/Country of Innovation/Published year	Inventors	Current assignee
Application of DPPA-entrapped AMD3100 nanoparticle in immunotherapy of residual cancer after liver cancer IRFA	CN117017948B/China/2025	Wenyue Zhang, Baoming Luo, Wanrong Luo, Pei’e Cai, Xiaojiang Chen	Sun Yat Sen Memorial Hospital Sun Yat Sen University
Plumbum Preparatium quinone lipid liquid crystal nanoparticle for treating liver cancer	CN121015563A/China/2025	Renjing JIA, Pengfei DU, Lu MOU, Jing YI, Kunying LIU	Chengdu University
A liver-targeted hypericin delivery nanocarrier and its preparation method and application	CN120437082A/China/2025	Qianqian Dang, Lin SUN, Xuelin WANG	Jilin University
Liver tissue imaging magnetic resonance contrast agent and preparation method and application	CN118304443A/China/2024	Mingyuan GAO, Jianfeng Zeng, Zang Li, Dandan Zhou, Kun Zhang	Suzhou Xin Ying Biological Medicine Technology Co ltd, Suzhou University
Preparation method and application of mixed charge gold nanoparticles	CN117357669A/China/2024	Jianjun Lai, Zhizeng Luo, Zhibing WU	Zhejiang Hospital
Sirna-copolymer compositions and methods of use for treatment of liver cancer	AU2022310516A1/Australia/2024	Patrick Lu, Michael MOLYNEAUX	Sirnaomics Inc
A nanocarrier for inhibiting tumor stemness and its preparation method and application	CN115990270A/China/2023	Zhenzhen Chen, Yalan Chen, Bingyu Li, Xueqin Zhu, Xiaoxi Wang, Wenyan Zhang, Yan Wang, Sijia Liu, Zimai Liu	Zhengzhou University
Antibody fragments conjugated to peg-plga nanoparticles improve immunotherapy against cancer cells	US20230086800A1/United States/2023	Sibu Kuruvilla, Dean W. Felsher, Christina Kim Lee	Leland Stanford Junior University
Dye-stabilized nanoparticles and methods of their manufacture and therapeutic use	US20200237670A1/United States/2020	Daniel A. Heller, Yosef SHAMAY	Memorial Sloan Kettering Cancer Center
Preparation method of anti-hepatocellular carcinoma nanoparticles loaded with two drugs and with double-layer controlled release-magnetic targeting-photothermal-magnetic thermal functions	CN109125293B/China/2020	Bing Wang, Xiaokang Jin, Biling Chen, Junmin Wan, Zhiwen Hu	Zhejiang University of Technology ZJUT

Recent clinical studies of nanoparticles for the diagnosis and treatment of HCC have been performed using ClinicalTrial.gov and are summarized in Table 3.

**TABLE 3:** An overview of clinical trial advancements in the diagnosis and management of HCC.

NCT number	Study title	Interventions	Phases	Enrollment	Study type
NCT07224464	The Sarah Nanotechnology System for Treatment of Advanced Metastatic Solid Tumors Using Hyperthermia	DEVICE: The Sarah Nanotechnology System-intended to deliver thermal energy to malignant cells for the purpose of thermal destruction of these cells in patients with advanced metastatic solid tumors	NA	9	INT
NCT06173466	Postoperative Analgesia With Liposomal Bupivacaine Versus Standard Bupivacaine Combined With Dexamethasone	DRUG: Liposomal bupivacaine DRUG: Bupivacaine Hydrochloride combined with dexamethasone	PHASE4	96	INT
NCT04791228	A Pilot Study of Thermodox and MR-HIFU for Treatment of Relapsed Solid Tumors	DEVICE: Magnetic Resonance-Guided High Intensity Focused Ultrasound DRUG: Lyso-thermosensitive Liposomal Doxorubicin	PHASE2	0	INT
NCT04682847	Radiotherapy With Iron Oxide Nanoparticles (SPION) on MR-Linac for Primary & Metastatic Hepatic Cancers	DRUG: Ferumoxytol injection	NA	40	OBS
NCT02721056	NBTXR3 Crystalline Nanoparticles and Stereotactic Body Radiation Therapy in the Treatment of Liver Cancers	RADIATION: NBTXR3, IL or IA injection + SBRT	PHASE1PHASE2	23	INT
NCT02527772	Liposomal Doxorubicin Plus Gemcitabine Versus Oxaliplatin Plus Fluorouracil/Leucovorin for Hepatocellular Carcinoma	DRUG: Liposomal Doxorubicin + Gemcitabine DRUG: FOLFOX4	PHASE2PHASE3	0	INT
NCT02181075	Targeted Chemotherapy Using Focused Ultrasound for Liver Tumours	DRUG: ThermoDox® (LTLD) DEVICE: Focused Ultrasound of Target Liver Tumour DIAGNOSTIC TEST: Pre-LTLD Biopsy of Target Liver Tumour| DIAGNOSTIC TEST: Post-LTLD Biopsy of Target Liver Tumour DIAGNOSTIC TEST: Post-LTLD + FUS (Post-FUS) Biopsy of Target Liver Tumour DEVICE: Thermometry of Target Tumour	PHASE1	10	INT
NCT02112656	Study of ThermoDox With Standardized Radiofrequency Ablation (RFA) for Treatment of Hepatocellular Carcinoma (HCC)	DRUG: ThermoDox DRUG: Dummy infusion	PHASE3	554	INT
NCT01829971	A Multicenter Phase I Study of MRX34, MicroRNA miR-RX34 Liposomal Injection	DRUG: MRX34	PHASE1	155	INT
NCT00617981	Phase 3 Study of ThermoDox With Radiofrequency Ablation (RFA) in Treatment of Hepatocellular Carcinoma (HCC)	DRUG: ThermoDox DRUG: 5% Dextrose Solution	PHASE3	701	INT
NCT00019630	Liposomal Doxorubicin in Treating Children With Refractory Solid Tumors	DRUG: doxorubicin HCl liposome	PHASE1	NA	INT
NCT00441376	A Study of ThermoDox in Combination With Radiofrequency Ablation (RFA) in Primary and Metastatic Tumors of the Liver	DRUG: ThermoDox	PHASE1	30	INT

OBS-observational; INT-interventional; NA-indicates missing date.

## 6 Perspectives and challenge

In conclusion, theranostic integration in HCC not only facilitates early diagnosis but also enhances therapeutic efficacy while minimizing systemic toxicity, thereby advancing HCC management and offering novel strategies to combat this malignancy. However, critical challenges persist. First, achieving precise nanomaterial targeting and optimizing tumor permeability remains a hurdle. Emerging solutions leverage nanotechnology advancements, including surface-engineered proteins, molecular targeting ligands, or biomimetic coatings (e.g., cell membranes, EVs), to enhance specificity. Subsequent permeability improvements rely on the EPR effect or externally triggered hyperthermia at tumor sites. Second, refining diagnostic and therapeutic precision requires multimodal approaches. Diagnostic accuracy can be elevated through complementary imaging modalities (e.g., MRI-PET-CT fusion), while therapeutic outcomes may be amplified via combined regimens (e.g., chemo-photodynamic therapy). The growing development of nanoplatforms capable of simultaneous multimodal imaging and combinatorial therapy provides a robust foundation for HCC theranostics, accelerating the translation of promising preclinical studies into clinical practice.
